# Self-Reported Satisfaction to Treatment, Quality of Life and General Health of Type 2 Diabetes Patients with Inadequate Glycemic Control from North-Eastern Romania

**DOI:** 10.3390/ijerph18063249

**Published:** 2021-03-21

**Authors:** Elena-Daniela Grigorescu, Cristina-Mihaela Lăcătușu, Ioana Crețu, Mariana Floria, Alina Onofriescu, Alexandr Ceasovschih, Bogdan-Mircea Mihai, Laurențiu Șorodoc

**Affiliations:** 1 Diabetes, Nutrition and Metabolic Diseases, “Grigore T. Popa” University of Medicine and Pharmacy, 700115 Iași, Romania; elena-daniela-gh-grigorescu@umfiasi.ro (E.-D.G.); alina.onofriescu@umfiasi.ro (A.O.); bogdan.mihai@umfiasi.ro (B.-M.M.); 2 Diabetes, Nutrition and Metabolic Diseases, “Sf. Spiridon” Emergency Hospital, 700111 Iași, Romania; 3 Department Preventive Medicine and Interdisciplinarity, “Grigore T. Popa” University of Medicine and Pharmacy, 700115 Iași, Romania; 4 Internal Medicine Clinic, Emergency Military Clinical Hospital, 700483 Iași, Romania; floria.mariana@umfiasi.ro; 5 Internal Medicine, “Grigore T. Popa” University of Medicine and Pharmacy, 700115 Iași, Romania; alexandr.ceasovschih@umfiasi.ro (A.C.); laurentiu.sorodoc@gmail.com (L.Ș.); 6 Department of Internal Medicine, Clinical Emergency Hospital “Sf. Spiridon”, 700106 Iași, Romania

**Keywords:** type 2 diabetes, quality of life, satisfaction to treatment, quality-adjusted life years, DTSQ, ADDQoL-19, SF-36, SF-6D

## Abstract

Type 2 diabetes mellitus (T2DM) undermines health and quality of life (QoL). This cross-sectional study surveyed 138 consenting T2DM patients from North-Eastern Romania with regard to their satisfaction with treatment, diabetes-related impact on QoL, and general health. The Romanian versions of Diabetes Treatment Satisfaction Questionnaire (DTSQ), Audit of Diabetes Dependent Quality of Life (ADDQoL-19), and 36-Item Short Form Health Survey (SF-36) questionnaires were used. Self-reports were analyzed in conjunction with clinical and metabolic profiling. The patients were 57.86 ± 8.82 years old, 49.3% men, treated with oral glucose-lowering drugs, presenting with inadequate glycemic control but without cardiovascular manifestations. The mean DTSQ and ADDQoL scores were 25.46 ± 0.61 and −2.22 ± 1.2, respectively. Freedom to eat, holidays, journeys, leisure, physical health, sex life, freedom to drink, and feelings about the future scored below average. The mean SF-36 physical and mental health scores were 47.78 ± 1.03 and 50.44 ± 1.38, respectively. The mean SF-6D score was 0.59 ± 0.04 (generated retrospectively using SF-36 data). Negative associations were significant between ADDQoL, age (r = −0.16), and body mass index (r = −0.23), *p* < 0.01. Overall scores did not correlate with diabetes duration (except DTSQ, r = −1.18, *p* = 0.02) or HbA_1c_. The results confirm other researchers’ findings in Europe and nearby countries. Our patients seemed satisfied with treatment despite glycemic imbalance and viewed diabetes as a burden on QoL and especially freedom to eat.

## 1. Introduction

Type 2 diabetes has become an international public health emergency and one of the great challenges of our time due to its prevalence and complexity. It is currently the most common chronic metabolic disorder worldwide: a staggering ~463 million adults of all ages are suffering from diabetes, according to the latest reports by the International Diabetes Federation (IDF). By 2030, the IDF hypothesizes that 195 million adults over the age of 65 will be diagnosed with diabetes [1]. In Romania, a nation-wide study indicated that 11.6% of the population had type 2 diabetes at the end of 2014. The estimated prevalence in the North-Eastern region specifically was 12.38%, higher than the national average [2].

By now, numerous original studies and subsequent reviews on the epidemiology, mechanisms, and complications of diabetes are available. They help inform new therapeutic approaches and generate paradigmatic shifts [3]. For instance, innovative medication was developed to address the chronic hyperglycemia caused by the so-called “Ominous Octet” of physiological and pathological abnormalities: insulin resistance in the muscles, liver and adipose tissue, progressive β-cell failure and apoptosis, increased α-cell secretion of glucagon concurrent with heightened hepatic sensitivity to it, β-cell resistance to glucagon-like peptide-1 and glucose-dependent insulinotropic polypeptides, elevated renal tubular glucose reabsorption, and altered neurotransmitter dysfunction. Mitigating chronic hyperglycemia is of utmost importance because, if left unresolved, it eventually triggers serious cardiovascular manifestations and systemic dysfunctions with potentially devastating effects [4]. Justifiably, the international medical community is invested in a concerted effort to deepen understanding, optimize treatments, and reduce the risks of cardiovascular and other complications [5].

For patients, diabetes is an insidious illness they must learn to live with long-term. For healthcare professionals, clinical aspects must be considered in relation to the patients’ unique personal contexts and attributes. This is also illustrated in the terminology: some authors use “PWD” with the meaning of “person/people with diabetes” rather than strictly “patient(s)” [6]. A diagnosis of diabetes typically engages the person in a complex process of (self-) education, guided therapy, monitoring, lifestyle changes, possibly even redefined life goals. This is made additionally challenging by the ubiquitous presence of some triggers and aggravating factors in everyday environments (e.g., foods containing added sugars).

Apart from experiencing the clinical features of diabetes, its complications, treatments, and restrictions, the mere idea of disease, too, can add an unwelcome layer of complexity. It can burden and cloud patients’ mindset with negative thoughts and feelings; these may further undermine their condition and ability to enjoy life [1,7]. Therefore, the relationship between objective clinical characteristics and subjective perspectives is worth exploring. We can then prepare for more effective encounters with our patients, tailoring medical advice and/or the method of delivery with increased awareness of the pitfalls and challenges of self-managing diabetes.

While clinicians have acquired advanced expertise to establish diagnoses and therapeutic goals, to achieve and monitor metabolic control, more can be done to acknowledge and address non-medical aspects. An integrated approach includes exploring, monitoring, and actively facilitating patients’ quality of life from the moment of diagnosis, during treatment, and throughout diabetes-related complications or other health-altering conditions/events [7,8].

Research into self-reported health issues now commonly includes quality of life (QoL) as an outcome, although the concept is an elusive one, inherently subject to the variability of individual experience and perspective [9,10,11]. There is no unanimous definition of quality of life and no single methodology to measure it with undisputed objectivity. In diabetes research, the heterogeneity of QoL survey tools encumbers the systematic review of how people with diabetes appraise their overall condition [12,13,14]. For instance, there is a certain degree of semantic overlap between quality of life and psychological well-being. Notably, some authors have attempted to outline a framework to help us discern the most relevant QoL measures for the PWD population [12,15,16,17]. Despite methodological challenges, the attention that recent clinical guidelines is beginning to pay to health-related QoL is an obvious improvement [7]. The International Consortium for Health Outcomes Measurement (ICHOM) has defined a set of Patient-Reported Outcomes, e.g., well-being, diabetes distress, and depression, arguing that their inclusion in diabetes research is a necessity, not a matter of preference (see ICHOM—standard set Variables for diabetes in adults<measuring results that matter) [18]. Diabetes-related quality of life is, we agree, a key psychosocial topic of interest. Alongside the prevention of complications, enhancing QoL should be at the core of multifactorial patient care [7,15,19].

Systematic analyses have already connected the dots between poorer quality of life and more severe cases of diabetes (treated with insulin and layered with complications and comorbidities). Given that glycemic control was shown to correlate with QoL only weakly or insignificantly, focusing on HbA_1c_ levels and other biological parameters is not enough. Rather, clinicians should be encouraged to incorporate quality of life as an explicit topic and objective in standard care. Increased adherence to treatment and better outcomes overall can be achieved by empowering patients to address their unique combinations of factors and, as much as possible, to enjoy life on their own terms.

Concurrently, there is also a growing interest in methodologies for evaluating the wider economic costs and implications beyond the more obvious health care costs related to diagnoses and treatments. With each individual person whose quality of life is made poorer by chronic illness, many different types of consequences—not all tangible or obvious—add up to eventually burden an entire socioeconomic system. In the new millennium, health care authorities recognize the practical importance of measurements that can go beyond dry lifespan data (mortality, morbidity) to include information about the quality of life and to allow the calculation of cost-effectiveness in resource allocation. One such indicator is quality-adjusted life-years (QALY), which can be conveniently derived using certain scoring algorithms and questionnaire-based survey tools (SF-36 and SF-6D) [20,21,22].

Our research subscribes to these broader considerations, and our aim with this study is to better understand how type 2 diabetes patients from the North-Eastern region of Romania subjectively appraise their satisfaction with treatment and their quality of life. Given the scarcity of such published data from Romania, the results may contribute to the geographical comprehensiveness of research conducted by our peers from other countries. This study is part of a doctoral research project aiming to assess the effects of certain therapeutic interventions on subclinical inflammation and cardiac function in type 2 diabetes patients with no clinical signs of atherosclerotic cardiovascular disease [23]. The data we present and discuss on this occasion reflect the authors’ appreciation for patients’ humanity and for holistic medical approaches.

## 2. Materials and Methods

This is a single-center, prospective, observational study of type 2 diabetes patients seen regularly at the Clinical Center for Diabetes, Nutrition and Metabolic Diseases in Iași, NE Romania. The study was designed in accordance with the ethical recommendations of the 1975 Helsinki Declaration, and it was formally approved both by the Research Ethics Committee of the “Grigore T. Popa” University of Medicine and Pharmacy Iași, and by the Ethics Committee of the “Sf. Spiridon” Emergency Clinical Hospital also in Iași (no. 63274/16.12.2015).

**Figure 1:**
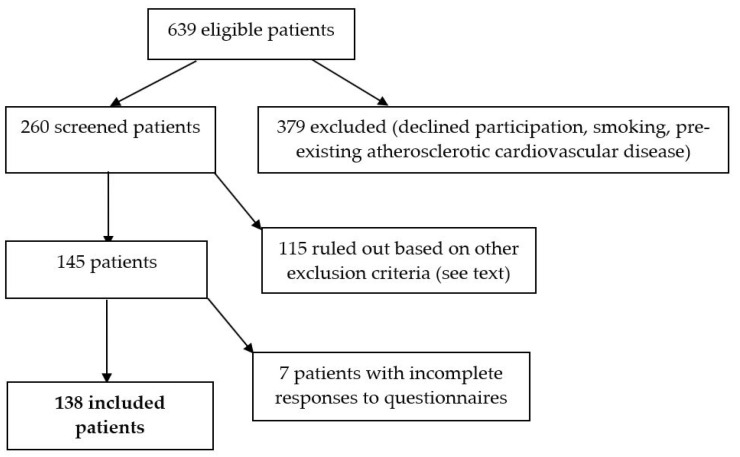
Overview of the patient enrollment process.

During June 2016–February 2018, of the diabetes patients attending our Clinical Center 639 were recommended by their diabetologist to add incretin-based drugs in order to optimize treatment. It is these patients who were contacted and invited to participate in the study; 74 declined participation, mainly on financial grounds (travel expenses). Previously known medical and social history resulted in the exclusion of another 305 cases (193 subjects had atherosclerotic cardiovascular disease, and 112 were smokers). The remaining consenting patients were invited for a planned one-day admission consisting of a battery of tests and interviews. Another 115 were excluded after preliminary screening (47 with uncontrolled blood pressure, 19 with ischemia findings on the electrocardiogram, 34 with increased triglycerides) (see Figure 1).

Clinically, we sought to attain comprehensive knowledge of the patients’ metabolic profiles, to screen for any diabetes-related chronic complications, and to adjust therapeutic plans accordingly (hence the need for functional hepatic and renal tests as well).

We collected anthropometric data (including weight and height for calculating the body mass indices BMI (kg/m^2^) = weight (kg)/(height (m))^2^, background information about the patients’ past medical histories, and about their present condition. We performed standard clinical examinations, blood tests, and urine sample tests, thus learning about the patients’ glycemic and lipid profiles, levels of glycated hemoglobin HbA_1c_, insulin, C-peptide, high sensitivity C-reactive protein (hs CRP), and uric acid. HbA_1c_ measurement was performed by DCCT aligned ion-exchange high-performance liquid chromatography using Bio-Rad *D-10™.* The immunological measurements were determined with chemiluminescence techniques (IMMULITE 1000). The left ventricular systolic and diastolic functions were assessed via transthoracic echocardiography and in accordance with the newest recommendations [24]. In addition, 12-lead ECGs were performed. 

To obtain the patients’ subjective appraisal of their condition, we chose a combination of three well-known questionnaires dealing with satisfaction to treatment, perceived quality of life, and self-assessment of overall health [25,26,27].

The criteria for inclusion in this study were as follows: (a) written informed consent; (b) age between 35 and 80; (c) prior diagnosis of type 2 diabetes according to the definition and classification by the World Health Organization (WHO) [28]; and (d) uncontrolled (HbA_1c_ > 7%) despite prior therapy with metformin and/or sulphonylurea or acarbose, making the patient eligible for further treatment adjustments such as the introduction of incretin-based medication. Patients were excluded on the basis of (a) disinterest, inability, or unwillingness to consent with the study, as well as poor compliance with the research process; (b) diagnosis of type 1 diabetes or secondary pancreatic diabetes; (c) insulin-based treatment of type 2 diabetes; (d) one or more of the following: satisfactory HbA_1c_ < 7%, elevated triglycerides >400 mg/dL, uncontrolled blood pressure (BP) >140/90 mmHg; (e) presence of atherosclerotic cardiovascular disease (myocardial infarction, angina, coronary revascularization, electrocardiogram findings of ischemia, stroke, transient ischemic attack, peripheral arterial disease) or valvular heart disease, mitral annular calcification, dysrhythmias, use of a cardiac pacemaker; (f) past medical history of inflammatory and severe acute or chronic conditions (pancreatitis, liver failure, gastrointestinal and kidney diseases, malignancies); (g) psychiatric disorders; (h) pregnancy or intention to become pregnant, and (i) smoking or recent history of smoking (in withdrawal for <1 year). 

### 2.1. Methodology for Collecting Patient-Reported Outcomes

We opted for the Diabetes Treatment Satisfaction Questionnaire (DTSQ) because of its wide application, validation, and translation to over 100 languages since its development in the 1990s. The DTSQ is also approved by the International Diabetes Federation and by the World Health Organization. It is very efficient, including only 8 items of which 6 relate to one’s treatment experience, and an additional 2 items refer specifically to the perceived burden of hyper-/hypoglycemic episodes. Answers are provided on a 0–6 Likert-type scale of satisfaction and convenience (maximum score 36). We used the status version of DTSQ [25,29,30].

For assessing the quality of life, we chose the already validated Romanian translation of ADDQoL—The Audit of Diabetes-Dependent Quality of Life 19 (the updated version with 19 items). Available in more than 20 languages, this is another popular survey tool among clinicians and researchers interested in evaluating the patients’ perceived overall impact of diabetes on their life [14,17]. The questionnaire invites respondents to consider a broad spectrum of life domains: leisure activities, working life, local/long journeys, holidays, physical health (things one could physically do), family life, friendship and social life, personal (closest) relationship, sex life, physical appearance, self-confidence, motivation, people’s reactions, feelings about the future, financial situation/status, living conditions, dependence on others, freedom to eat, and freedom to drink. The patients indicate to what degree their life would be better or worse in the absence of diabetes. Details on ADDQoL scoring are found in ref. [31].

The DTSQ and ADDQoL-19 were applied with the consent and license received from Claire Bradley from the Health Psychology Research, Department of Psychology, Royal Holloway, University of London (https://www.healthpsychologyresearch.com, accessed on 1 November 2015). The license of the Romanian language version was marked with the reference number CB 513/11.11.2015.

Thirdly, SF-36 (Short Form 36 v2) is a free and validated questionnaire of 36 items used to assess one’s perceived state of health across 8 scales considering general, physical, mental, and social functions and difficulties. This tool was not designed specifically for patients with diabetes, but we were nevertheless interested in our patients’ perspectives and in using SF-36 as a corroboration tool for ADDQoL. The Romanian version chosen was the outcome of a standardized translation and validation process guided by the creators of the original English version from New England Medical Center in the US [27,32]. The patients’ responses to SF-36 were also used retrospectively to calculate the SF-6D score because that would allow us to consider the patients’ quality of life also from the perspective of public health and economics. Cost and utility analyses can be done by measuring quality-adjusted life years (QALY). Basically, SF-6D provides a way to use the SF-36 data to calculate QALY, thus gaining an integrated view of longevity and quality of life (values range from 0 meaning death to 1 meaning perfect health). SF-6D is a multi-attribute utility instrument that uses a standardized health state descriptive system of six domains: physical functioning, role limitation, social functioning, pain, mental health, and vitality. In the literature, the SF-6D classification system appears to be continually updated, and there is growing interest in developing a protocol value set for international application in research on both the general population and specific patient cohorts [33,34,35,36]. We used the second version of the questionnaire (SF-6Dv2) and the value set model 3 in Table 3 provided by Mulhern et al. to calculate health utility scores [33,36].

### 2.2. Statistical Analysis

The statistical analysis was performed using IBM SPSS Statistics for Windows (version 17, SPSS Inc, Chicago, IL, USA). All tests were two-tailed, and a *p*-value < 0.05 was considered as statistically significant. Baseline characteristics were expressed as frequencies, percentages, means, standard deviations, medians, and interquartile ranges (IQR) taking into consideration distributions and types of variables (quantitative or qualitative data). The reliability of the ADDQoL questionnaire was checked by calculating Cronbach’s Alpha coefficients. Measures of associations between nominal variables were studied using Phi and Cramer’s V coefficients. Parametric and non-parametric tests (ANOVA, Kruskal–Wallis, Mann–Whitney U) were used to compare means or medians (questionnaire scores) between specific groups (i.e., stratified by gender, presence of neuropathy). Spearman correlation coefficients were calculated to identify significant associations between variables without normal distribution.

## 3. Results

### 3.1. General and Clinical Characteristics of the Study Cohort

Of all the diabetic patients presenting at the diabetes center during the specified period, 138 met the inclusion criteria for this study. The mean age of the participants was 57.86 ± 8.82, and 55.8% were between 50 and 64 years old. In addition, 68 of them were men (49.3%). The mean value for the body mass index (BMI) was 32.65 ± 5.50 kg/m^2^, with abnormal waist circumference in all but three cases (97.8%). Obesity (BMI ≥ 30 kg/m^2^) was noted in approximately 66% of cases.

Regarding the patients’ known history of type 2 diabetes, 45.7% had been diagnosed within the previous 5 years, while 26.1% had been living with the disease for over 10 years (see Table 1). Upon enrollment, all patients had their HbA_1c_ levels >7%, HbA_1c_ exceeded 7.5% in two-thirds of cases. Additionally, 68% presented levels of triglycerides >150 mg/dL, 44.2% had hypercholesterolemia (with LDL-cholesterol > 70 mg/dL in 82.6% of cases), and a third were hyperuricemic. 

Having already excluded patients with certain comorbidities and complications (see exclusion criteria), some patients still presented sensorimotor peripheral neuropathy (42.20%) and mild forms of non-proliferative diabetic retinopathy (10 cases). Additionally, only a minority of patients (12 cases) did not suffer from other comorbidities such as non-alcoholic steatohepatitis (75.46% of cases), dyslipidemia (71.74%), and arterial hypertension (67.4%, all with BP values maintained under 130/80 mmHg by antihypertensive therapy). Grade I diastolic dysfunction was detected by echocardiography in 83 patients, using the latest available guideline for the evaluation of diastolic function. 

The antihyperglycemic therapy consisted of a single oral drug in 73.18% of cases (98 patients were on metformin) and in two drugs in 23.18% of cases (32 patients were on metformin and sulphonylurea). Only 5 patients were taking a combination of metformin, sulphonylurea, and acarbose. Some patients were on hypertensive and hypolipemiant medication to manage their comorbidities (56 on statins, 35 on diuretics, 39 on angiotensin-converting enzyme inhibitors, 37 on beta-blockers, 26 on calcium channel blockers, and 23 on angiotensin-II-receptor antagonists). 

### 3.2. Patient-Reported Outcomes

Regarding the patients’ level of satisfaction with the treatment, we calculated a mean DTSQ score of 25.46 ± 0.61 relative to the maximum possible of 36. The answers ranged from 3 (the lowest) to 36 (the highest), and the median value was 26 (IQR 10). The aspects rated highest by most patients were their understanding of the disease, their satisfaction with their treatment, its perceived convenience and flexibility, as well as their interest in continuing to adhere to it. Half of the patients gave high scores indicating an unfavorable attitude towards the frequency of hyperglycemic episodes, while only 8% of them found the frequency of hypoglycemic episodes unacceptable (see Table 2). We found an inverse relationship between the mean DTSQ score and patients’ finding high glucose unacceptable (r = −0.18, *p* = 0.03), concurrent with a positive association between the DTSQ score and the rating of low glucose as unacceptable (r = 0.2, *p* = 0.01). In addition, 45.7% of patients signaled their willingness to recommend their treatment to others by scoring the respective item between 4 and 6 (a median answer of 3 across the entire cohort).

When DTSQ results were correlated to the patients’ demographic and clinical characteristics, we noticed higher mean scores from men (26.59 ± 6.37) than women (24.36 ± 7.85), even though gender and satisfaction did not significantly correlate (*p* = 0.06). Patients with BMIs >30 kg/m^2^ reported significantly less satisfaction to treatment than those with lower BMIs (24.55 vs. 27.71, *p* = 0.01). When using Phi and Cramer’s V association coefficient, above-average satisfaction with treatment associated significantly with the duration of diabetes (−0.25 at *p* = 0.011), but not with the degree of glycemic imbalance present in all patients (0.127, *p* = 0.33). Moreover, the patients with a duration of diabetes of less than 5 years reported significantly higher levels of satisfaction to treatment compared both to those who had been suffering from the disease for 5–10 years (27.25 vs. 23.95, *p* = 0.02) and for those diagnosed more than 10 years prior (27.25 vs. 23.94, *p* = 0.02). Patients who experienced sensorimotor polyneuropathy appeared relatively less satisfied than those who did not (24.10 vs. 26.53, *p* = 0.04); the presence of other complications and comorbidities did not correlate significantly with lower DTSQ scores.

Regarding the patients’ appraisal of their quality of life using the ADDQoL questionnaire, we checked first for reliability and found that the internal consistency was strong (Cronbach’s Alpha coefficient was 0.92). The overall Average Weighted Impact (AWI) score was −2.22 ± 1.2 (median value −2.21, IQR 1.76), with a majority of 73 patients providing mean scores of −2.21 or less. The overall AWI score was 0, as in no impact, in only 5 cases. In response to the two introductory questions, 68.4% of patients appreciated their quality of life as good to excellent (median −1), but only 8.7% would have given the same scores had they not been suffering from diabetes. In fact, 50.7% indicated that their quality of life would be much better in the absence of diabetes.

**Figure 2:**
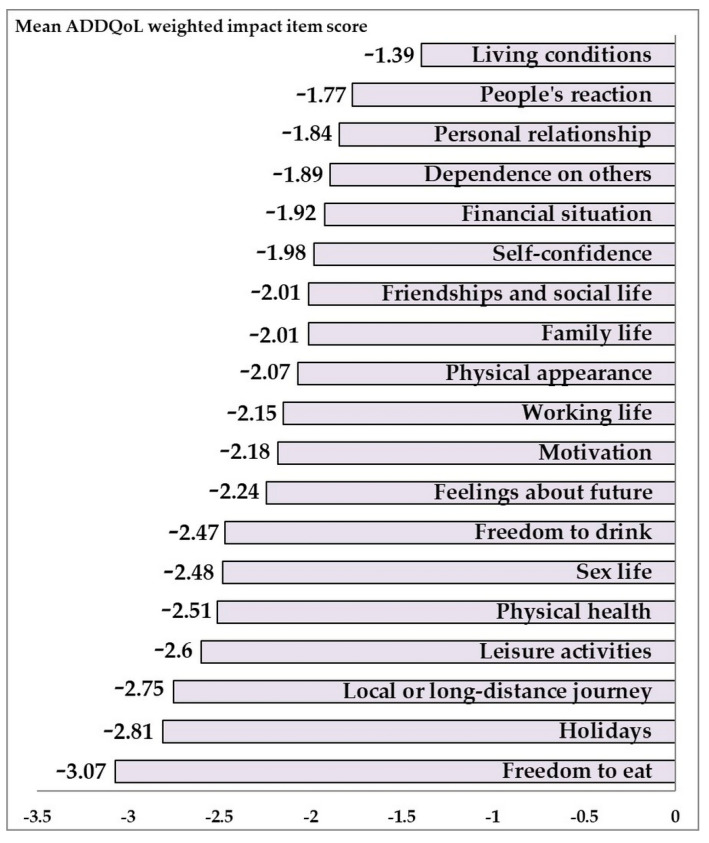
The mean weighted impact (ADDQoL scores) of diabetes on individual life domains in the population study.

The more specific views resulting from the questionnaire are summarized in Table 3. We also included in the table the numbers of patients who abstained from rating domains that they thought did not apply to their situation (e.g., working life, holidays, family life, close personal relationships, or sex life). Freedom to eat was found to be the aspect most strongly influenced by diabetes (AWI score −3.07 ± 2.09). Other negative impacts were holidays (AWI score −2.81 ± 1.81), local or long-distance journeys (AWI score −2.75 ± 2.08), and leisure activities (−2.6 ± 1.63). Living conditions and people’s reaction were the two domains reported as the least negatively impacted by diabetes (AWI scores −1.39 ± 2.33 and −1.77 ± 2.16, respectively) (see Figure 2).

The statistical processing of the ADDQoL scores in relation to the patients’ objective characteristics revealed a significant association with gender (an overall AWI score of −1.99 ± 1.31 for men vs. −2.44 ± 1.05 for women; *p* = 0.03). Diabetes seemed to exert a more noteworthy negative impact on women across all life domains except the freedom to drink, although some of the corresponding score differences did not reach statistical significance (items 5–9, 13 and 14: physical health, family life, friendships and social life, personal relationship, sex life, people’s reactions, feelings about the future). 

Age showed an overall negative association with AWI (r = −0.16 at *p* < 0.01). In addition, patients under 50 and older than 65 reported significantly greater negative impact of diabetes on aspects addressed by items 11 and 13–16 (self-confidence, people reaction, feelings about the future, financial situation/status, living condition). The patients’ overall AWI scores ranged inversely proportional in relation to their BMIs (r = −0.23 at *p* < 0.01), but few of the other clinical characteristics correlated significantly with the self-reported QoL scores. Neither the duration of diabetes and the level of glycemic imbalance, nor the presence of complications and comorbidities, and not even the number of medications taken seemed to significantly diminish the patients’ general view of their quality of life. For instance, patients who had been living with diabetes for over 10 years reported a more negative overall impact of the illness (−2.41 ± 1.18) compared to those with a more recent diagnosis (−2.35 ± 1.22 for 5–10 years of diabetes and −2.03 ± 1.19 for <5 years of diabetes). However, statistical significance was not present (*p* > 0.05). A statistically significant *p*-value was reached only for perceived impact on local or long-distance journeys by patients with more recent diabetes (<5 years) and those with over 10 years of diabetes (−2.39 vs. −3.27, *p* = 0.02), and physical appearance by patients with more recent diabetes (<5 years) versus those with 5–10 years of diabetes (−1.74 vs. −2.48 at *p* = 0.028).

**Figure 3:**
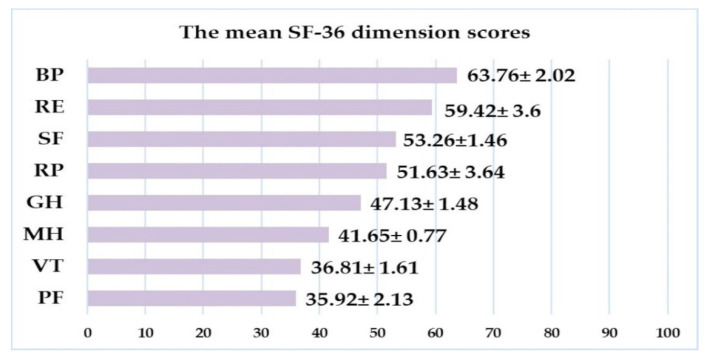
The mean scores for the SF-36 dimensions. PF, physical functioning; VT, vitality; MH, mental health; GH, general health; RP, role-physical; SF, social functioning; RE, role-emotional; BP, bodily pain. Range 0–100, higher scores indicate more favorable views of own health.

Next, the answers to the SF-36 v2 questionnaire revealed that, overall, the physical component score PCS had a mean value of 47.78 ± 1.03 relative to the maximum possible value of 100 (median 49.25, IQR 19.94), and the mental component MCS had a mean value of 50.44 ± 1.38 (median 51.15, IQR 27.71). For mean SF-36 dimensions scores, see Figure 3. The application of the Spearman formula revealed statistically significant correlations between the patients’ appraisal of their quality of life (overall AWI score to ADDQoL), physical health (PCS), and their mental health (MCS) (r = 0.27, *p* = 0.001, respectively, r = 0.30, *p* < 0.001, respectively). When we analyzed the PCS and MCS values for subgroups based on certain variables (age, sex, diabetes duration, or presence of neuropathy), only the physical component was scored differently by men compared to women (54.03 for men vs. 46.85 for women at *p* = 0.01). Last but not least, the SF-36 data were used retrospectively to calculate utility scores with SF-6D v2. The main result was that SF-6D scores were normally distributed and had a mean value of 0.591 ± 0.044. As can be seen in Table 4, no significant differences were found with regard to diabetes duration, age, or sex (even if the highest values were noted in men).

## 4. Discussion

First, with regard to the inclusion of outpatients only, it is worth pointing out that, at our clinic, we strive to encourage patient self-management and only hospitalize emergencies. As such, most of our work consists of outpatient care, and most diabetes patients come in regularly for follow up and adjustments to their treatments. Based on clinical experience, we know that hospitalized patients suffering from severe and/or complicated manifestations find their quality of life very strongly undermined by their condition. In addition, regarding cohort size, our explicit interest was in the diabetes patient whose condition is advancing (hence the inclusion criterion for inadequate glycemic control) but not yet severe or complicated to the point where its detrimental effects on the patient’s quality of life would be self-evident (hence some of the exclusion criteria). In addressing the challenges mentioned in the Introduction, these patients represent an important demographic niche: with adequate understanding and support, they could avert or delay aggravated manifestations. We return to these points in the sub-section on limitations.

Another methodological discussion point concerns our choice of questionnaires and the decision to use several. We began by surveying the literature looking to select topic-appropriate, well-performing, standardized questionnaires that were (1) reasonably short and easy to fill in, (2) complementary with regard to the relevant issues, and (3) widely used, especially in Europe and neighboring countries. This was done to facilitate patient participation, obtain comprehensive and reliable data, and compare results internationally [10,12,15,17,37,38,39,40,41,42,43,44,45].

### 4.1. Satisfaction to Treatment (DTSQ)

Regarding the patients’ satisfaction with their treatment, we found that patients with a longer history of diabetes, higher BMI, and/or polyneuropathy were less satisfied. Though more than two-thirds of the patients (76.79%) reported high levels of satisfaction with items 1, 4, and 8, half were also surprisingly unbothered by their perceived hyperglycemia. This result goes against the inversely proportional relationship between the objectively measured glycemic control (HbA_1c_) and reduced satisfaction to treatment found by other studies [40]. At the same time, we also saw a statistically significant, inverse relation between lower satisfaction with treatment and frequent hyperglycemia.

The discrepancy between our results and other studies may be due, at least in part, to how conversations unfolded between the patients and their attending clinician with regard to the next steps in the treatment: it is possible that the patients found them psychologically reassuring. At the time of participating in our survey, some patients were waiting for approval by the National Health Insurance Authority to start incretin-based treatment. This used to be a required bureaucratic step. Updating the therapeutic plan to add incretin or another innovative drug could only be solicited upon demonstrable glycemic imbalance (HbA_1c_ > 7%). At present, the process has been simplified, and national protocols allow the introduction of innovative drugs, taking into consideration newly available studies regarding cardiovascular benefits (including sodium-glucose cotransporter-2 inhibitors and glucagon-like peptide-1 receptor agonist) [3].

The significant, positive association between our patients’ satisfaction with treatment and perceptions of low glucose levels as unacceptable is also noteworthy. Nicolucci et al. reported a similar result. One possible explanation has been put forth by Boels: the episodes of hypoglycemia could be perceived as the price the patient needs to pay in order to keep their glucose level from going up and avert the ensuing consequences. It sounds reasonable, but our study cannot contribute substantially to verifying this point because too few of our patients were exposed to the risk of hypoglycemic events due to medication [40,46].

Such varied results regarding how patients assess their glycemic levels and satisfaction to treatment may be good news for clinicians seeking to boost patient satisfaction because it helps patients comply. Basically, in the absence of severe complications and insulin treatment, satisfaction to treatment does not appear to depend too strictly on achieving clinical targets.

Last but not least, relative to the DTSQ results, it was interesting to see that the patients held favorable views regarding their treatment (mean 25.46 ± 0.61 out of the maximum value 36 in DTSQ) while also appraising negatively the impact of diabetes on their quality of life. This brings us to a discussion of the ADDQoL results.

### 4.2. Quality of Life (ADDQoL and SF-36)

The overall ADDQoL mean score shows, as expected, that diabetes does exert a negative impact on the patients’ perceptions of their quality of life. More than 50% of participants indicated that their lives would be better in the absence of diabetes. We also observed a negative association with age, found also by Papazafiropoulos (who additionally found that living alone correlated with lower scores) [47].

Equally noteworthy is that we did not find significant correlations between the overall self-reported outcomes and the patients’ objectively measured HbA_1c_. This is not new in the literature: other studies using the same instruments indicated that patients do not necessarily see a meaningful connection between glycemic control and their quality of life. For example, Kuznetsov et al. conducted their study on 1876 patients from Denmark, the Netherlands and the UK upon 5 years following the diagnosis and found that 60% of the patients with HbA_1c_ < 7% treated with oral antidiabetic drugs gave higher scores to ADDQoL (−0.32) and SF-36 (PCS 46.2 and MCS 54.6) [35]. However, when adjusting for age, gender, BMI, intake of glucose-lowering drugs and education level, the multivariate analysis did not yield statistically significant associations between the patients’ HbA1c values and their subjective assessments. Similarly, Arditi et al. found no significant correlation with the objectively measured glycemic control in their cross-sectional analysis of 585 patients responding to ADDQoL and SF-36 [48].

One challenge in reaching a definitive conclusion is that the patients enrolled in relevant studies were quite diverse in terms of age, type and history of diabetes, treatment plans, comorbidities, and complications. Different authors also used different cutoff limits for glycemic imbalance. Even so, systematic reviews and meta-analyses confirm that patients associate worse QoL-related outcomes with a longer history of diabetes, the presence of complications, and of hypertension. Concurrently, physical exercise and frequent monitoring of glucose levels correlate with better QoL consistently across 18 studies on approximately 57,000 patients [49]. Additionally, in a study using the SF-36 questionnaire on 1352 T2DM patients, the multivariate analysis adjusted for illness duration and other risk factors showed that patients with HbA_1c_ < 6.9% and those with HbA_1c_ > 8.6% provided significantly different assessments for both physical and mental health components (the higher the HbA_1c_, the lower the PCS and MCS scores) [50]. The authors of another interventional study found that intensive lifestyle interventions could, one year on, positively impact patients’ appraisal of physical health (PCS), but not necessarily mental health as well (MCS). The patients in question were, on average, 53.6 years old and had a history of T2DM of 4.5 years [51]. In our study, the lowest reported SF-36 subscale score was for physical functioning (and vitality), and the highest was for bodily pain. Our mean PCS and MCS values were similar to those reported by clinical trials investigating the cardiovascular and subjective outcomes of new medication [52].

However, compared to other European studies using ADDQOL scores to assess diabetes-related overall QoL, the Romanian participants in our study rated theirs as poorer (−2.2) compared to other groups, such as from Switzerland and Slovenia (−1.6) [48,53], but similar to patients from Greece and Bulgaria (−2.9) [45,47,54]. The SF-6D index derived from the SF-36 responses was also lower than in other studies [20,22,55,56].

This adds to existing evidence of complex differences across regions. Among the factors which may explain such differences, we can hypothesize that the provision of higher standards of care in some countries (e.g., Switzerland) may have an appeasing, reassuring, psychological effect on the patients living there [48].

From the detailed ADDQoL results, we found that freedom to eat was the most negatively impacted aspect. This is in accordance with the majority of published studies, such as the European-wide Panorama study on a total of 5817 T2DM patients from nine countries (Belgium, France, Germany, Greece, Italy, the Netherlands, Spain, Turkey, and the UK). In this multinational research, freedom to eat featured as the most negatively impacted. The results also point to the complexity of the therapeutic plan (e.g., three drugs, insulin) as a predictor for the subjective appraisal of one’s quality of life as significantly poorer. However, compared to our study, the Panaroma research enrolled patients who were, on average, older (65.9), less overweight (54.4% with a BMI < 30 kg/m^2^), with a longer history of diabetes (8.9 years), with a lower incidence of poor glycemic control (37.4% with HbA_1c_ > 7%), and with treatment plans involving several drugs (only 32.6% were on a single antihyperglycemic agent). In addition, 24.5% were already diagnosed with macrovascular complications, unlike the patients in our sample, all of whom did not present clinical atherosclerotic manifestations [41].

In addition, a subgroup analysis on 375 Greek patients showed higher satisfaction with treatment than in the case of our patients (DTSQ 29.1 ± 5.6) and a slightly more favorable overall ADDQoL score of AWI −2 ± 1.9. The aspects most negatively affected were, again, the freedom to eat, as in the case of our patients, but also the freedom to drink. The Greek study included a homogenous cohort gender-wise (51.1% women) but notably older than ours (average age 63.5), and with a longer history of diabetes (9.7 years). Most of the Greek patients were on a single-drug therapeutic plan, had unsatisfactory glycemic control (62.6% with HbA_1c_ > 7%), and elevated LDL-cholesterol (55.8%) [54].

It is well known that nutrition goals are of paramount importance in managing type 2 diabetes, especially in the case of overweight patients. Healthcare professionals should know the extent to which patients value their freedom to eat as a part of their quality of life. Dietary restrictions and, in many cases, reduced calorie intake, are challenging to establish and maintain, so counseling and education interventions are needed. This also implies adequate information and training for professionals. Successfully caring for patients with diabetes relies on effective strategies for long-term adherence to healthy eating plans adapted to both clinical targets and personal preferences [57].

Questionnaires such as DTSQ, ADDQoL, and SF-36 are helpful tools for investigating the connections between clinical, biological, and psychosocial patient characteristics [58,59]. They are also being validated in more and more countries for use with specific populations (e.g., Poland, Lithuania) [43,60]. In choosing them, we subscribe to internationally coherent data collection efforts and support the inclusion of Romanian patient experience in future meta-analyses. Importantly, studies such as ours and those referenced here can pave the way for prospective/interventional research and educational/training programs featuring QoL as an endpoint [43,59,60,61]. We also recognize that individual patient perspectives thus surveyed can be processed further to evaluate the wider economic implications of chronic illness. Public health experts and health economists have every reason to conduct such research into diabetes, given the worldwide prevalence of this illness, the daily burdens related to treatment and lifestyle, and the progressively detrimental impact on lifespan and the quality of life.

### 4.3. Limitations

Apart from the relatively small cohort size, which was already discussed, our study does not consider the socioeconomic characteristics of the patients, such as their level of education, profession, or income. It also does not provide a comparison with similar data from adequately controlled diabetic patients, or severe/complicated cases.

In part, this is due to the nature of our research interests. We explicitly wanted to focus on compliant, cooperating patients whose diabetes was serious but not complicated or severe enough to require complex treatments or hospitalization (which can be expected to have a marked negative impact on the quality of life). At the same time, many of the patients that we see on a regular basis already exhibit comorbidities, diabetes-related complications, or unhealthy habits such as smoking. This is, unfortunately, one of the realities of diabetes in Romania.

In addition, from a methodological standpoint, our experience with continued patient care as well as this research is that patients can easily tire of answering questions and filling in questionnaires. At the same time, we were concerned that a single, simple questionnaire, even if carefully drafted and standardized, would not provide enough coverage and reliability. Similarly, without ruling out important comorbidities, we could not isolate the influence of diabetes in answers which could be attributed to multiple conditions [15,17,62]. A year after the data collection period, even the patients who agreed to participate in this study preferred to maximize their subsequent appointments by having clinical tests and results (e.g., echocardiography) rather than answering more/repeated questionnaires. Any personal concerns relevant to the quality of life were simply brought up in conversation with the clinician, which for scientific research would be too anecdotal. Respondent fatigue is the main reason why we were unable to collect another batch of self-reported data from the same patients, even if we have since amassed the clinical information for a longitudinal analysis [63]. Last but not least, the observational design of this study prevents us from advancing any cause–effect explanations with regard to the observed relationships between objectively measured glycemic control and patient-reported outcomes.

## 5. Conclusions

Multiple studies have shown that dietary restrictions cause substantial inconvenience for diabetic patients on non-insulin treatment plans and without apparent cardiovascular complications. This study points to a similar trend in patients from NE Romania, who seemed most negatively affected by impositions on their freedom to eat. Our patients also reported wide-ranging and generally negative influences with regard to other life domains (e.g., living conditions and relationships), although to a lesser degree. Practitioners may use such information in order to anticipate the patients’ difficulty in adhering to dietary recommendations as an important part of their diabetes self-management.

## Figures and Tables

**Table 1:** Patients’ characteristics.

Characteristic	Total (N = 138)	Variables	Value
Age, years *	57.86 (8.82)	Glycemia (mg/dL) **	162 (46)
**Gender, N (%)**		**Insulin (** µIU/mL) **	11.2 (9.39)
Women	70 (50.7)	C-peptide (ng/mL) **	3.26 (2.22)
Men	68 (49.3)	hs-CRP (mg/dL) **	5.35 (9.18)
Diabetes duration, years **	5 (8)	HbA_1c_ (%) **	7.8 (1.12)
<5 (N, %)	63 (45.7)	<7.5 (N, %)	46 (33.3)
5–10 (N, %)	39 (28.2)	7.5–8 (N, %)	42 (30.4)
≥10 (N, %)	36 (26.1)	≥8 (N, %)	50 (36.3)
BMI (kg/m^2^) *	32.65 (5.50)	Total cholesterol (mg/dL) *	195.33 (46.14)
**Hypertension, N (%)**	93 (67.39)	HDL-cholesterol (mg/dL) *	56.79 (15.27)
**Dyslipidemia, N (%)**	99 (71.74)	LDL-cholesterol (mg/dL) *	103.12 (38.96)
**Steatohepatitis, N (%)**	104 (75.46)	Triglycerides (mg/dL) *	202.57 (90.46)
**Neuropathy, N (%)**	61 (44.20)	Uric acid (mg/dL) *	5.48 (1.43)
**Retinopathy, N (%)**	10 (7.24)	eGFR (mL/min/1.73 m^2^ *	82 (16.37)
**Diastolic dysfunction, N (%)**	83 (60.14)	ACR (mg/g) *	27.14 (48.64)

BMI, body mass index; hs-CRP, high-sensitivity C-reactive protein; HDL, high-density lipoprotein; LDL, low-density lipoprotein; eGFR, estimated glomerular filtration rate; ACR, albumin-to-creatinine ratio; * Values are expressed as the mean (SD, standard deviation); ** Values are expressed as the median (IQR, interquartile range); N, number.

**Table 2:** Diabetes Treatment Satisfaction Questionnaire results—as assessed by patients.

Items of DTSQ’s Specific Domains	Scores *	% of Patients with Positive Answers **
1. Current treatment satisfaction	4.46 ± 0.12 5 (2)	79
2. Unacceptable high glucose	3.23 ± 0.15 3.5 (3)	50
3. Unacceptable low glucose	1.02 ± 0.12 0 (2)	8
4. Convenience	4.72 ± 0.11 5 (2)	84
5. Flexibility	4.42 ± 0.13 5 (3)	76.8
6. Understanding	4.42 ± 0.12 5 (3)	72.5
7. Willing to recommend	2.85 ± 0.21 3 (6)	45.7
8. Satisfaction to continue	4.58 ± 0.13 5 (2)	76

* Values are expressed as means ± standard deviation and median (interquartile range); ** positive answers: range 4–6 in DTSQ (patient scored each item on a scale ranging from 0 “very dissatisfied/inconvenient” to 6 “very satisfied/convenient”); the total score was calculated as the sum of the scores for items 1, 4, 5–8.

**Table 3:** Patients’ responses to the Romanian version of the Audit of Diabetes Dependent Quality of life (ADDQoL-19).

**Specific Life Domain**	**Impacting Rating**	**Importance Rating**	**Weighted Impact Score**
Leisure activities	−1.86 ± 0.91	2.12 ± 0.64	−2.60 ± 1.73
Working life (34) *	−1.26 ± 0.95	1.91 ± 0.73	−2.15 ± 1.85
Local or long-distance journeys	−1.34 ± 0.94	2.20 ± 0.74	−2.75 ± 2.08
Holidays (18) *	−1.37 ± 0.91	2.24 ± 0.73	−2.81 ± 1.81
Physical health	−1.37 ± 0.83	2.01 ± 0.69	−2.51 ± 1.55
Family life (1) *	−1.36 ± 0.96	1.58 ± 0.63	−2.01 ± 1.57
Friendship and social life	−1.10 ± 0.97	2.03 ± 0.66	−2.01 ± 1.83
Personal relationship (14) *	−1.14 ± 1.01	1.74 ± 0.68	−1.84 ± 1.69
Sex life (25) *	−1.29 ± 0.97	2.12 ± 0.81	−2.48 ± 2.04
Physical appearance	−1.15 ± 0.98	2.03 ± 0.62	−2.07 ± 1.68
Self-confidence	−1.20 ± 0.93	1.72 ± 0.63	−1.98 ± 1.65
Motivation	−2.18 ± 1.82	2.00 ± 0.65	−1.20 ± 0.99
People’s reaction	−0.89 ± 1.00	2.33 ± 0.88	−1.77 ± 2.16
Feelings about feature	−1.33 ± 1.01	1.93 ± 0.70	−2.24 ± 1.87
Financial situation	−1.05 ± 1.04	1.96 ± 0.77	−1.92 ± 2.12
Living condition	−0.74 ± 1.04	1.91 ± 0.72	−1.39 ± 2.33
Dependence on others	−0.99 ± 1.03	1.98 ± 0.88	−1.89 ± 2.10
Freedom to eat	−1.62 ± 1.01	2.15 ± 0.80	−3.07 ± 2.09
Freedom to drink	−1.20 ± 1.06	2.49 ± 0.86	−2.47 ± 2.22

Notes. The values are reported as means ± standard deviation. Impact rating (condition without diabetes mellitus): −3, very much better; −2, much better; −1, a little better; 0, the same; +1, worse; Importance rating: 0, not all important; 1, somewhat important; 2, important; 3, very important.; Weighted impact score = impact rating (−3 to +1) x importance rating (0 to 3) = −9 (maximum negative impact of diabetes) to +3 (maximum positive impact of diabetes); * Number of patients with no available response.

**Table 4:** SF-6Dv2 utility scores according to the patients’ characteristics

Characteristics	Total (N = 138)	SF-6Dv2 (Mean, 95% CI)	*p* Value
Age (years) *	57.86 ± 8.82	0.591 (0.583–0.598)	0.883
<50	30 (21.7)	0.591 (0.571–0.610)	
50–64	77 (55.8)	0.592 (0.582–0.602)	
≥65	31 (22.5)	0.587 (0.583–0.598)	
**Gender, N (%)**			0.415
Women	70 (50.7)	0.588 (0.577–0.598)	
Men	68 (49.3)	0.594 (0.582–0.605)	
Diabetes duration, years **	5 (8)		0.249
<5 (N, %)	63 (45.7)	0.585 (0.574–0.596)	
5–10 (N, %)	39 (28.2)	0.600 (0.584–0.616)	
≥10 (N, %)	36 (26.1)	0.590 (0.577–0.603)	
BMI (kg/m^2^) *	32.65 (5.50)		0.94
<24.9 (N, %)	6 (4.35)	0.584 (0.557–0.612)	
25–29.9 (N, %)	41 (29.71)	0.591 (0.575–0.606)	
≥30 (N, %)	91 (65.94)	0.591 (0.582–0.600)	
HbA_1c_ (%) **	7.8 (1.12)		0.946
<7.5 (N, %)	46 (33.3)	0.589 (0.576–0.602)	
7.5–8 (N, %)	42 (30.4)	0.591 (0.577–0.605)	
≥8 (N, %)	50 (36.3)	0.592 (0.583–0.598)	
**With Neuropathy, N (%)**	61 (44.2)	0.592 (0.582–0.602)	0.696
**Without Neuropathy N (%)**	77 (55.8)	0.578 (0.578–0.600)	
**With Hypertension, N (%)**	93 (67.4)	0.591 (0.582–0.599)	0.931
**Without Hypertension, N (%)**	45 (32.6)	0.590 (0.575–0.605)	
**With Dyslipidemia, N (%)**	99 (71.7)	0.595 (0.586–0.604)	0.064
**Without Dyslipidemia, N (%)**	39 (28.3)	0.579 (0.565–0.594)	
DTSQ **	26 (10)		0.346
≤26, **N (%)**	71 (51.4)	0.587 (0.577–0.598)	
>26, **N (%)**	67 (48.6)	0.594 (0.583–0.605)	
ADDQoL **	−2.21 (1.76)		0.787
≤−2.21, **N (%)**	73 (52.9)	0.592 (0.581–0.603)	
>−2.21, **N (%)**	65 (47.1)	0.590 (0.579–0.600)	

SF-6Dv2, Short Form Six Dimensions version 2; DTSQ, Diabetes Treatment Satisfaction Questionnaire; ADDQoL, Audit of Diabetes Dependent Quality of life; BMI, body mass index; * Values are expressed as the mean (SD, standard deviation); ** Values are expressed as the median (IQR, interquartile range); N, number; CI, confidence interval.
